# Supplementary material for: Publication Trends in Neglected Tropical Diseases of Latin America and the Caribbean: A Bibliometric Analysis
Source: Pathogens. 2021 Mar 17;10(3):356. doi: 10.3390/pathogens10030356 (PMC8002643; doi:10.3390/pathogens10030356)
Supplement: Supplementary file 1 [file pathogens-10-00356-s001.pdf]

	Country	Buruli ulcer	Chagas disease	Chromomycosis	Cysticercosis	Dengue	Zika	Chikungunya	Hydatid cyst	Fascioliasis	Leishmaniasis	Leprosy	Lymphatic filariasis	Mycetoma	Onchocerciasis	Rabies	Schistosomiasis	Soil Transmitted Helminthiasis	Trachoma	Yaws	Malaria	HIV/AIDS	Tuberculosis
1	United States	113	2197	31	354	5096	4118	1594	261	136	2618	887	871	74	543	1323	1910	842	2099	55	16322	22041	16485
2	Brazil	5	3978	131	140	2173	1596	651	87	84	4574	1237	66	35	57	303	1183	271	150	3	2258	1661	2993
3	Mexico	3	506	27	190	557	171	143	25	36	235	76	3	43	26	73	14	41	31	2	258	305	999
4	Colombia	2	438	7	3	539	287	229	5	16	405	53		1	4	32	9	52	24		713	183	458
5	Argentina		1357	2	14	230	89	60	155	78	246	21	6	4	9	26	22	89	52		137	255	534
6	Canada	4	201	4	27	380	309	106	70	28	524	107	52		58	207	194	103	166	3	1471	2345	2236
7	Peru	3	125	4	171	154	52	24	48	59	190	8	1	1	4	27	9	64	21		346	168	586
8	Venezuela		235	2	15	132	59	47	2	8	150	15	2	1	9	2	20	33	6		139	35	101
9	Chile		320	1	6	31	29	13	77	21	42	11	2	3		29	6	7	15		73	126	195
10	Ecuador		100		50	83	57	45	4	7	67	7	2		8	3	5	42	2		69	19	60
11	Guatemala		48	1	1	17	19	8		1	5				21	6	1	4	5		40	18	24
12	Cuba		30	1	1	169	16	5	2	26	73	11				5	3	10	4	1	46	39	81
13	Bolivia		172		2	22	17	20	2	5	51	3			1	1	2	18	1		33	4	20
14	Haiti					12	8	11				1	40		2	23	3	3	3		89	55	73
15	Dominican Republic		1	1		21	11	20		2			8	1		4	1	1			18	19	13
16	Honduras		15		2	24	27	8			6	1						11	1		26	16	13
17	Paraguay		82			38	7	7		1	45	2				1		4	2		6	6	17
18	Nicaragua		13		6	135	67	27			2	1						6			7	1	
19	El Salvador		17			17	7	5	2		2	1				1		1	2		9	5	8

20	Costa Rica		15	1		42	10	13			21					5		1	1	40	8	19
21	Panama		55		1	51	18	22	1		50			1	1	6		2	1	99	7	34
22	Puerto Rico (US)[5]																					
23	Uruguay		225		9	55	27	9	90	48	51	1	1			6	31	1		25	25	74
24	Jamaica		1			18	10	14				1					1	2	1	16	34	17
25	Trinidad and Tobago		1		2	42	21	18			1	2	1			8				9	6	17
26	Guyana		1			1	3				2		2			1				13	3	6
27	Suriname		1			4	12	4			8	3				1			6	40		3
28	Guadeloupe (France)																					
29	Martinique (France)																					
30	Bahamas						1														7	
31	Belize					3	1	1												9	2	1
32	Barbados					13	3	3												5	5	2
33	French Guiana (France)	3	12	1			36	29	1		32	5	1	1		7	1	5	1	104	14	16
34	Saint Lucia							1									3			3	2	2
35	Curaçao (Kingdom of the Netherlands)					2	3	8												1		1
36	Aruba (Kingdom of the Netherlands)						1	5										1			1	
37	Saint Vincent and						1														1	

	the Grenadines														
38	United States Virgin Islands (US)														
39	Grenada	2	9	5	15	8	2		4	4	3		9	8	4
40	Antigua and Barbuda										1		1	1	3
41	Dominica		2	2	2					1					
42	Bermuda (UK)														
43	Cayman Islands (UK)		2	1	1									1	
44	Greenland (Denmark)														
45	Saint Kitts and Nevis		8		4	1	5	1	20	6	6			2	2
46	Sint Maarten (Kingdom of the Netherlands)		1												
47	Turks and Caicos Islands (UK)													1	
48	Saint Martin (France)				2							1		2	
49	British Virgin Islands (UK)														
50	Caribbean Netherlands (Kingdom of the Netherlands)												1		1

51	Anguilla (UK)	2	7
52	Saint Barthélemy (France)		
53	Saint Pierre and Miquelon (France)		
54	Montserrat (UK)		
55	Falkland Islands (UK) [6]		
